# Biomedical Text Categorization Based on Ensemble Pruning and Optimized Topic Modelling

**DOI:** 10.1155/2018/2497471

**Published:** 2018-07-22

**Authors:** Aytuğ Onan

**Affiliations:** Celal Bayar University, Department of Software Engineering, 45400 Turgutlu, Manisa, Turkey

## Abstract

Text mining is an important research direction, which involves several fields, such as information retrieval, information extraction, and text categorization. In this paper, we propose an efficient multiple classifier approach to text categorization based on swarm-optimized topic modelling. The Latent Dirichlet allocation (LDA) can overcome the high dimensionality problem of vector space model, but identifying appropriate parameter values is critical to performance of LDA. Swarm-optimized approach estimates the parameters of LDA, including the number of topics and all the other parameters involved in LDA. The hybrid ensemble pruning approach based on combined diversity measures and clustering aims to obtain a multiple classifier system with high predictive performance and better diversity. In this scheme, four different diversity measures (namely, disagreement measure,* Q*-statistics, the correlation coefficient, and the double fault measure) among classifiers of the ensemble are combined. Based on the combined diversity matrix, a swarm intelligence based clustering algorithm is employed to partition the classifiers into a number of disjoint groups and one classifier (with the highest predictive performance) from each cluster is selected to build the final multiple classifier system. The experimental results based on five biomedical text benchmarks have been conducted. In the swarm-optimized LDA, different metaheuristic algorithms (such as genetic algorithms, particle swarm optimization, firefly algorithm, cuckoo search algorithm, and bat algorithm) are considered. In the ensemble pruning, five metaheuristic clustering algorithms are evaluated. The experimental results on biomedical text benchmarks indicate that swarm-optimized LDA yields better predictive performance compared to the conventional LDA. In addition, the proposed multiple classifier system outperforms the conventional classification algorithms, ensemble learning, and ensemble pruning methods.

## 1. Introduction

The immense quantity of biomedical text documents can serve as an essential source of information for biomedical research. Biomedical text documents are characterized by an immense quantity of unstructured and sparse information in a wide range of forms, such as scientific articles, biomedical datasets, and case reports. Text mining aims to identify valuable information from unstructured text documents with the use of tools and techniques from several disciplines, such as machine learning, information retrieval, and computational linguistics. The use of text mining is one of the most promising tools in the biomedical domain that has attracted a lot of research interest. Text mining in biomedical domain can be successfully applied in a wide range of applications, including identification of disease-specific knowledge [1], diagnosis, treatment, and prevention of cancer [2], identification of obesity status of patients [3], identification of risk factors for heart disease [4], annotation of gene expression [5], and identification of drug targets and candidates [6].

Biomedical text mining follows the same stages (namely, format conversation, tokenization, stop word removal, normalization, stemming, dictionary construction, and vector space construction) utilized in the text processing from other domains [7]. To build accurate classification schemes on text documents, one pivotal issue is to identify an appropriate representation model for the documents [8]. The vector space model (also known as term vector model) is one of the most commonly employed representation schemes to process text documents, owing to its simple structure [9]. In this model, each text document is represented as vectors of identifiers (index terms). The vector space model suffers from high dimensional feature space, irrelevancy, and sparsity of features. Since each document is represented as a bag of words with the corresponding frequencies, words are regarded as statistically independent. Hence, word order is not taken into consideration [10].

Considering the limitations of the vector space model and the high dimensional unstructured nature of biomedical text documents, there are a number of representation schemes (such as the latent semantic analysis, the probabilistic latent semantic analysis, and the latent Dirichlet allocation) employed to process biomedical text documents [7]. The latent semantic analysis (LSA) is a scheme to extract and represent the contextual meaning of words with the use of statistical computations utilized on a large amount of text [11]. LSA can represent the semantic relations within the text. It can find the latent classes, while reducing the dimensionality of vector space model [12]. However, LSA has no strong statistical foundation and can suffer from high mathematical complexity [13]. The probabilistic latent semantic analysis (PLSA) is a statistical method for analysis of data which is based on a latent class model. PLSA has a strong statistical foundation. It can find latent topics and it can yield better performance compared to LSA [13].

The latent Dirichlet allocation (LDA) is an efficient generative probabilistic topic model, where each document is represented as a random mixture of latent topics. LDA can find latent topics, reduce the high dimensionality of vector space model, and can outperform other linguistic representation schemes, such as latent semantic analysis and probabilistic latent semantic analysis [14]. LDA involves several parameter values, such as the number of topics, the number of iterations for Gibbs sampling, *α* parameter to control the topic distribution per document, and *β* parameter to model distributions of terms per topic (Panichella et al., 2003). For unstructured text documents, information about the document-wise content and number of relevant topics is not known in advance (Zhao et al., 2005). Hence, the identification of an appropriate value for the number of topics is a challenging problem for unstructured text documents. An insufficient or excessive number of topics can degrade the predictive performance of machine learning algorithms built on LDA-based topic modelling. In addition to the number of topics, LDA requires several other parameters. Therefore, finding an optimal configuration for LDA-based topic modelling involves extensive empirical analysis with different configurations.

In order to build robust classification schemes, multiple classifier systems (also known as ensemble classifiers) have been widely employed in the field of pattern recognition, owing to its remarkable improvement in generalization ability and predictive performance [15]. There are three main stages of the ensemble learning process, namely, ensemble generation, ensemble pruning, and ensemble combination [16, 17]. The ensemble generation stage is the phase, in which base learning algorithms to be utilized in the multiple classifier system are generated. The base learning algorithms can be generated either homogeneously or heterogeneously. The ensemble combination stage seeks to integrate the individual predictions of base learning algorithms. The ensemble pruning stage aims to identify an optimal subset of base learning algorithms from the ensemble to enhance the predictive performance and computational efficiency. It has been empirically validated that ensemble pruning can yield more robust classification schemes [18].

Considering these issues, we propose a multiple classifier approach to biomedical text categorization based on swarm-optimized topic modelling and ensemble pruning. In the presented scheme, swarm-optimized approach is employed to estimate the parameters of LDA, including the number of topics and all the other parameters involved in LDA. Motivated by the success of hybrid ensemble pruning schemes [19–21], the proposed approach combines diversity measures and clustering. In this scheme, four different diversity measures (namely, disagreement measure,* Q-*statistics, the correlation coefficient, and the double fault measure) are computed to capture the diversities within the ensemble. Based on these diversity measures, a combined diversity matrix is obtained. Based on this matrix, a swarm intelligence based clustering algorithm partitions the classification algorithms into a number of disjoint groups and one algorithm (with the highest predictive performance) from each cluster is selected to build the multiple classifier system. In the empirical analysis, five biomedical text benchmarks have been utilized. In the swarm-optimized LDA, different metaheuristic algorithms (such as genetic algorithms, particle swarm optimization, firefly algorithm, cuckoo search algorithm, and bat algorithm) are considered. In addition, five different metaheuristic clustering algorithms are considered in the ensemble pruning stage. The empirical analysis on biomedical text benchmarks indicates that swarm-optimized LDA yields better predictive performance compared to the conventional LDA. In addition, the proposed hybrid ensemble pruning scheme outperforms the conventional classification algorithms and ensemble learning methods.

The main contributions of our proposed categorization scheme can be summarized as follows:

- We introduced a metaheuristic approach to optimize the set of parameters utilized in LDA-based topic modelling. In this regard, the number of topics (*k*), the number of Gibbs iterations (*n*), *α* parameter to control the topic distribution per document, and *β* parameter to model distributions of terms per topic are considered. We conducted several experiments on swarm-optimized LDA with different metaheuristic algorithms (namely, genetic algorithms, particle swarm optimization, firefly algorithm, cuckoo search algorithm, and bat algorithm). To the best of our knowledge, this is the first comprehensive empirical analysis devoted to metaheuristic algorithms on LDA-based topic modelling.
- We introduced an ensemble pruning approach based on combined diversity measures and metaheuristic clustering. To the best of our knowledge, this is the first study in ensemble pruning, which utilizes metaheuristic clustering algorithms to obtain diversified base learning algorithms.
- The presented classification scheme, which integrates swarm-optimized LDA-based modelling with the hybrid ensemble pruning scheme, is employed on biomedical text categorization. To the best of our knowledge, this is the first comprehensive study on LDA-based topic modelling and ensemble pruning on biomedical text categorization.

The rest of this paper is structured as follows. In Section 2, related work on topic modelling and multiple classifier systems have been presented.  Section 3 presents the theoretical foundations, Section 4 presents the proposed text categorization framework, Section 5 presents the experimental results, and Section 6 presents the concluding remarks.

## 2. Related Work

This section presents the related work on topic modelling and multiple classifier systems in biomedical text categorization.

### 2.1. Related Work on Topic Modelling

Topic modelling models have been successfully employed to summarize large-scale collections of text documents. Probabilistic topic modelling methods can be utilized to identify the core topics of text collections. In addition, topic modelling schemes can be utilized in a variety of tasks in computational linguistics, such as analysis of source code documents [23], summarizing opinions of product reviews [24], identification of topic evolution [25], aspect detection in review documents [26], analysis of Twitter messages [27], and sentiment analysis [28, 29].

Probabilistic topic modelling has attracted the attention of researchers on biomedical domain. Biomedical text collections suffer from high dimensionality and topic modelling methods are effective tools to handle with large-scale collections of documents. Hence, topic modelling can yield promising results on biological and biomedical text mining [30]. For instance, Wang et al. [31] presented a probabilistic topic modelling scheme to identify protein-protein interactions from the biological literature. In this scheme, the correlation between different methods and related words is modelled in a probabilistic way to extract the detection methods. In another study, Arnold et al. [32] utilized the latent Dirichlet allocation method to identify relevant clinical topics and to structure clinical text reports. Song and Kim [33] employed the latent Dirichlet allocation method to conduct bibliometric analysis on bioinformatics from full-text text collections of PubMed Central articles. In another study, Sarioglu et al. [34] utilized topic modelling to represent clinical reports in a compact way, so that these collections can be efficiently processed. In another study, Bisgin et al. [35] applied topic modelling to drug labelling, which is a human-intensive task with many ambiguous semantic descriptions. In this way, manual annotation challenges can be eliminated. Likewise, Wang et al. [36] introduced a topic modelling based scheme to identify literature-driven annotations for gene sets. In this scheme, the number of topics to be utilized in topic modelling is empirically inferred through the analysis with various parameter values (5, 10, 15, 20, etc.) for the number of topics. In another study, Bisgin et al. [37] employed the latent Dirichlet allocation based topic modelling to identify interdependencies between cellular endpoints. The experimental analysis indicated that LDA can substantially enhance the understanding of systems biology. Probabilistic topic modelling has also been employed to identify drug repositioning strategies [38]. Wang et al. [39] utilized topic modelling to analyze 17,723 abstracts from PubMed publications related to adolescent substance use and depression. In this study, topic modelling was employed to identify the literature and to capture other relevant topics. In another study, Wang et al. [40] presented a topic modelling based scheme to mine biomedical text collections. In this scheme, topic modelling was employed as a fine-grained preprocessing model. Recently, Sullivan et al. [41] utilized topic modelling to identify unsafe nutritional supplements from review documents. In another study, Chen et al. [42] employed probabilistic topic modelling to represent hospital admission processes in a compact way.

### 2.2. Related Work on Multiple Classifier Systems

Multiple classifier systems have been successfully employed in a wide range of applications in pattern recognition, including biomedical domain. Empirical analysis on multiple classifier systems indicates that ensemble pruning can enhance the predictive performance of multiple classifier systems [18]. Ensemble pruning approaches can be mainly divided into five groups, as exponential search, randomized search, sequential search, ranking-based, and clustering based methods [16]. Exponential approaches to ensemble pruning seek to examine all possible subsets of base learning algorithms within the multiple classifier system. For instance, Aksela [43] examined the predictive performance of several evaluation metrics (namely, correlation between errors,* Q*-statistics, and mutual information) in ensemble pruning. Randomized approaches to ensemble pruning aim to explore the search space of candidate classifiers with the use of metaheuristic algorithms. A wide range of metaheuristics, such as genetic algorithms, tabu search, and population based incremental learning, have been successfully utilized for ensemble pruning [44, 45]. For instance, Sheen and Sirisha [46] introduced an ensemble pruning scheme for malware detection based on harmony search. Likewise, Mendialdua et al. [47] utilized the estimation of distribution algorithm for ensemble pruning. In sequential search based methods, the search space of candidate classifiers has been explored in forward, backward, or forward-backward direction. For instance, Margineantu and Dietterich [48] introduced a sequential approach for ensemble pruning based on reduced error pruning with back-fitting. Similarly, Caruana et al. [49] presented a forward stepwise selection based approach for ensemble pruning. Recently, Dai et al. [50] introduced a reverse reduced error-based ensemble pruning algorithm based on subtraction operation. Ranking-based approaches to ensemble pruning aim to identify an optimal subset of classifiers based on a ranking obtained by a particular evaluation measure. For instance, Kotsiantis and Pintelas [51] presented a* t*-test based ranking scheme for ensemble pruning. More recently, Galar et al. [52] presented an ordering-based metric for ensemble pruning. Clustering based approaches to ensemble pruning partition the base learning algorithms of ensemble into clusters. For instance, Zhang and Cao [53] presented a spectral clustering based algorithm for ensemble pruning. In this scheme, the base learning algorithms were grouped into two clusters based on predictive performance and diversity. Then, one cluster of ensemble was pruned and one cluster of ensemble was retained as the pruned subset of classifiers.

### 2.3. Motivation and Contribution of the Study

As outlined in advance, probabilistic topic modelling methods are essential tools to identify hidden topics in large-scale collections of text documents. In order to enhance the performance of LDA, there are a number of extensions on the basic model. For instance, Griffiths and Tenenbaum [54] introduced a hierarchical latent Dirichlet allocation model. In this model, topic distributions are identified from hierarchies of topics, where each hierarchy is modelled by a nested Chinese restaurant process. Each node of tree corresponds to a particular topic, where each topic is associated with a distribution. In another study, Teh et al. [55] presented a hierarchical latent Dirichlet allocation scheme, in which parameter value for the number of topics is inferred through the use of posterior inference. Grant and Cordy [56] introduced a heuristic approach to estimate the number of topics in source code analysis. In another study, Panichella et al. [57] presented a genetic algorithm based scheme to identify optimal configurations for latent Dirichlet allocation. In this scheme, parameter set for topic modelling was estimated with the use of genetic algorithm. The presented scheme was employed on three different tasks of software engineering, namely, traceability link recovery, feature location, and software artifact labelling. Likewise, Zhao et al. [58] introduced a heuristic approach to estimate the appropriate number of topics for latent Dirichlet allocation. In this scheme, the appropriate number of topics is identified through the use of ratio for perplexity change. Recently, Karami et al. [59] presented a fuzzy approach to topic modelling. In this scheme, fuzzy clustering was employed to identify optimal number of topics.

In addition to the aforementioned five ensemble pruning approaches, hybrid methods have attracted research attention in the pattern recognition. Hybrid approaches to ensemble pruning seek to integrate several ensemble pruning paradigms. For instance, Lin et al. (2014) introduced a hybrid ensemble pruning algorithm which integrates k-means clustering and dynamic selection. Similarly, Mousavi and Eftekhari [60] presented a hybrid ensemble pruning scheme which integrates static and dynamic ensemble selection with NSGA-II multiobjective genetic algorithm. In another study, Cavalcanti et al. [21] presented a hybrid ensemble pruning algorithm based on genetic algorithm and graph coloring. In this scheme, several different diversity measures (such as* Q*-statistics, correlation coefficient, Kappa statistics, and double fault measure) are combined via a genetic algorithm. Similarly, Onan et al. [19, 20] introduced a hybrid ensemble pruning algorithm based on consensus clustering and multiobjective evolutionary algorithm. In this scheme, classifiers are assigned into clusters based on their predictive performance and the set of candidate classifiers are explored through the use of evolutionary algorithm.

Recent studies on topic modelling indicate that the identification of an appropriate parameter value for the number of topics is an essential task to build robust classification schemes. In addition, hybrid ensemble pruning schemes can outperform conventional classifiers, ensemble learning methods, and ensemble pruning methods. Through their potential use on text classification, the number of works that utilize metaheuristic algorithms to optimize parameters of LDA and the number of works that utilize ensemble pruning schemes are very limited. To fill this gap, this paper presents a classification scheme based on swarm-optimized topic modelling and hybrid ensemble pruning for text categorization.

## 3. Theoretical Foundations

This section summarizes the theoretical foundations of the study. Namely, the latent Dirichlet allocation method, swarm-based optimization algorithms, ensemble learning methods, ensemble pruning methods, cluster validity indices, and pairwise diversity measures are presented.

### 3.1. The Latent Dirichlet Allocation

The latent Dirichlet allocation model (LDA) is a widely employed generative probabilistic model to identify the latent topics in text documents [22]. In LDA, each document is represented as a random mixture of latent topics and each topic is represented as a mixture of words. The mixture distributions are Dirichlet-distributed random variables to be inferred. In this scheme, each document exhibits the topics in different proportions, each word in each document is drawn among the topics, and topics are chosen based on per-document distribution over topics [61]. LDA attempts to determine the underlying latent topic structure based on the observed data. In LDA, the words of each document correspond to the observed data. For each document in the corpus, words are obtained by following a two-staged procedure. Initially, a distribution over topics is randomly chosen for each word of the document [22]. In LDA, a word is a discrete data from a vocabulary indexed by {1, …, *V*}, a sequence of* N *words *w*=(*w*_1_, *w*_2_*,…, w*_*n*_), and a corpus is a collection of* M *documents denoted by* D=*{w_1_, w_2_,…, w_*M*_}. The generative process of LDA is summarized in Box 1.

**Figure 1:**
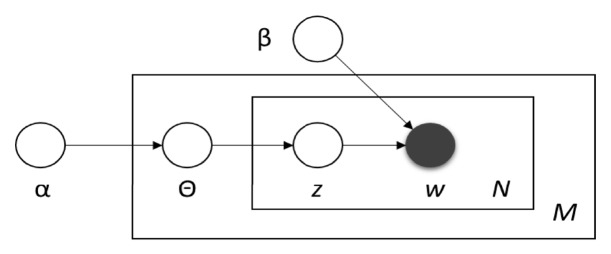
The graphical representation of LDA [22].

LDA process can be modelled by a three-level Bayesian graphical model, as given in Figure 1. In this graphical model, nodes are used to represent random variables and edges are used to denote the possible dependencies between the variables. In this notation, *α* refers to Dirichlet parameter, Θ refers to document-level topic variables,* z *refers to per-word topic assignment, *w* refers to the observed word, and *β* indicates the topics [61].

Based on this notation, the generative process of LDA corresponds to a joint distribution of the hidden and observed variables. The probability density function of a* k*-dimensional Dirichlet random variable is computed as given by (1), the joint distribution of a topic mixture is computed as given by (2), and the probability of a corpus is computed as given by (3) [22]:

(1) pΘ ∣ α=Γ∑i=1kαi∏i=1kΓαiΘ1α1−1…Θkαk−1

(2) pΘ,z,w ∣ α,β=pΘ ∣ α∏n=1Npzn ∣ Θpwn ∣ zn,β

(3) pD ∣ α,β=∏d=1M∫pΘd ∣ α·∏n=1Nd∑Zdnpzdn ∣ Θdpwdn ∣ zdn,βdΘd

In LDA, the computation of the posterior distribution of the hidden variables is an important inferential task. The exact inference of hidden variables is exponentially large. Hence, approximation algorithms (such as Laplace approximation, variational approximation, and Gibbs sampling) have been utilized in LDA process [61].

### 3.2. Ensemble Learning Methods

Ensemble learning methods aim to combine the predictions of multiple classification algorithms so that a classification model with higher predictive performance can be achieved [62]. In dependent methods, the outputs of former classifiers determine the outputs of following classifiers. In contrast, the outputs of classifiers are individually identified and combined to produce the final prediction in independent methods. Dependent ensemble methods include Boosting (e.g., AdaBoost algorithm) and independent methods include Bagging, Dagging, and Random Subspace. To examine the predictive performance of the proposed scheme, four well-known ensemble learning methods (namely, AdaBoost [63], Bagging [64], Random Subspace [65], and Stacking [66]) are considered.

### 3.3. Ensemble Pruning Methods

The ensemble pruning methods aim to identify optimal subset of classification algorithms to improve the predictive performance and computational efficiency of multiple classifier systems. To examine the predictive performance of proposed ensemble pruning algorithm, we have employed four ensemble pruning algorithms. These methods are the ensemble pruning methods from libraries of models [49], Bagging ensemble selection [67], LibD3C algorithm [68], and ensemble pruning based on combined diversity measures [21].

### 3.4. Swarm-Based Optimization Algorithms

Swarm-based optimization algorithms, including genetic algorithms, particle swarm optimization, firefly algorithm, cuckoo search algorithm, and bat algorithm, have been successfully employed on applications of data science, such as data clustering and data categorization [68]. In the proposed scheme, swarm-based optimization algorithms have been utilized to optimize the set of parameters of LDA-based topic modelling. In addition, the proposed ensemble pruning algorithm employs swarm-based optimization algorithms to group classifiers into clusters. In the empirical analysis, genetic algorithms [69], particle swarm optimization algorithm [70], firefly algorithm [71], cuckoo search algorithm [72], and bat algorithm [73] are utilized.

### 3.5. Cluster Validity Indices

This section briefly introduces four cluster validity indices (namely, the Bayesian information criterion, Calinski-Harabasz index, Davies-Bouldin index, and Silhouette index), which are utilized to evaluate the clustering quality of different configurations of LDA.

The Bayesian information criterion (BIC) is computed as given below:

(4) $$\begin{array}{c} BIC=-\ln\left(\;L\;\right)+vln\left(\;n\;\right) \end{array}$$

where* n *denotes the number of topics,* L *denotes the likelihood of parameters to generate data in the model, and* v *denotes the number of free parameters in Gaussian model [74]. The smaller the Bayesian information criterion, the better the generated model.

The Calinski-Harabasz index (CH) is the ratio of the traces of between cluster scatter matrix and the internal scatter matrix, which is computed as given below [74]:

(5) CHK=trace  B/K−1trace  W/N−K

(6) trace  B=∑k=1KCkck¯−x¯2

(7) trace  C=∑k=1K∑i=1Nwk,ixi−Ck¯2

where* K* denotes the number of clusters,* N *denotes the number of data instances, |*C*_*k*_| denotes the number of elements in cluster* C*_*k*_*, x*_*i*_ denotes a point within cluster* C*_*k*_,* B *denotes the between-cluster scatter matrix, which represents the error sum of squares between different clusters, and* W *denotes the internal scatter matrix, which represents the squared differences of instances in a cluster. Here, trace of an* n*-by-*n *square matrix corresponds to the sum of the elements on the main diagonal [75].

The Davies-Bouldin index (DB) is a cluster validity index, which aims to maximize between-cluster distance and to minimize the distance between centroids of clusters and the other data points, that is defined as given by the following equation:

(8) $$\begin{array}{c} BD=\frac{1}{c}\overset{c}{\underset{i=1}{\sum }}\underset{i\neq j}{\max}\left\{\;\frac{d\left(X_{i}\right)+d\left(X_{j}\right)}{d\left(c_{i},c_{j}\right)}\;\right\} \end{array}$$

where* c *denotes the number of clusters,* i *and* j *correspond to cluster labels,* d*(*c*_*i*_,* c*_*j*_) corresponds to distance between centroids of clusters, and *X*_i_ corresponds to a data point within cluster* C*_*i*_. The smaller the DB criterion, the better the generated model.

The Silhouette index (SI) is defined as given by (9):

(9) SI=1N∑i1ni∑x∈Cibx−axmax⁡bx,ax

(10) ax=1ni−1∑y∈Ci,y≠xdx,y

(11) bx=minj≠i⁡1ni∑y∈Cjdx,y

where* N *denotes the number of clusters, *n*_*i*_ denotes the size of cluster* C*_i_,* a*(*x*) denotes the average distance between the* i*th instance and all instances in* X*_j_*, b(x) *denotes the minimum distance from* i *to the centroids of clusters not containing* i. *

### 3.6. Pairwise Diversity Measures

This section briefly introduces four diversity measures (namely, disagreement measure,* Q-*statistics, the correlation coefficient, and the double fault measure) which are utilized in the proposed ensemble classification scheme.

*Q-*statistics, the correlation coefficient (*p*_*i,k*_), the disagreement measure (Dis), and the double fault measure (DF) among two classifiers* D*_*i*_ and* D*_*k*_ are computed using (12), (13), (14), and (15), respectively [76]:

(12) Qi,k=N11N00−N01N10N11N00+N01N10

(13) ρi,k=N11N00−N01N10N11+N10N01+N00N11+N01N10+N00

(14) Disi,k=N01+N10N11+N10+N01+N00

(15) DFi,k=N00N11+N10+N01+N00

where* N*^11^,* N*^00^,* N*^10^, and* N*^01^ denote the number of correctly classified instances by the two classifiers, the number of incorrectly classified instances by the two classifiers, the number of instances correctly classified by* D*_*i*_ and incorrectly classified by* D*_*k*_, and the number of instances correctly classified by* D*_*k*_ and incorrectly classified by* D*_*i*_, respectively.

## 4. The Proposed Text Categorization Framework

The proposed text categorization framework combines the swarm-optimized Latent Dirichlet allocation and diversity-based hybrid ensemble pruning scheme. The rest of this section explains the methods utilized in the proposed biomedical text categorization framework.

### 4.1. Swarm-Optimized Latent Dirichlet Allocation

The latent Dirichlet allocation (LDA) is an efficient generative probabilistic model that can be employed to represent unstructured text documents in an efficient way. In general, LDA-based topic modelling involves the calibration of several parameters, summarized as follows:

- Number of topics in LDA-based topic modelling (*k*).
- *α* parameter to control the topic distribution per document. A higher value for *α* parameter denotes better smoothing of topics for each document.
- *β* parameter to model distributions of terms per topic.

In order to improve the computational complexity of LDA, LDA is usually employed in conjunction with an approximation method. In this work, we utilized Gibbs sampling method in conjunction with LDA. In this way, the number of iterations (*N*) for sampling is also involved as an additional parameter value. Identifying appropriate parameter values of LDA with the optimal configuration is a challenging task. Without setting appropriate parameter values, LDA-based representation may degrade the predictive performance of classification schemes. Too low or too much number of topics can result in a poor predictive performance. Hence, finding an optimal configuration for LDA-based topic modelling involves extensive empirical analysis. Exhaustively enumerating possible parameter values for LDA to identify an optimal configuration involves high computational analysis with a wide range of parameter values.

In this paper, five metaheuristic algorithms (namely, genetic algorithms, particle swarm optimization, firefly algorithm, cuckoo search algorithm, and bat algorithm) are utilized to calibrate the parameters of LDA. In this scheme, values of all parameters of LDA are taken into consideration. Hence, various values for each parameter are evaluated to find an optimal configuration. In the presented problem, the first issue is to examine the merit of a particular LDA-based configuration. In order to evaluate the merit of a particular configuration of LDA before employing on a particular task, we have employed four internal cluster validity indices, namely, the Bayesian information criterion, Calinski-Harabasz index, Davies-Bouldin index, and Silhouette index. Higher clustering quality of a particular LDA-based configuration tends to yield higher predictive performance on LDA-based categorization tasks [19, 20]. For this reason, we seek to identify an LDA configuration which maximizes the overall clustering quality of LDA configuration.

**Figure 2:**
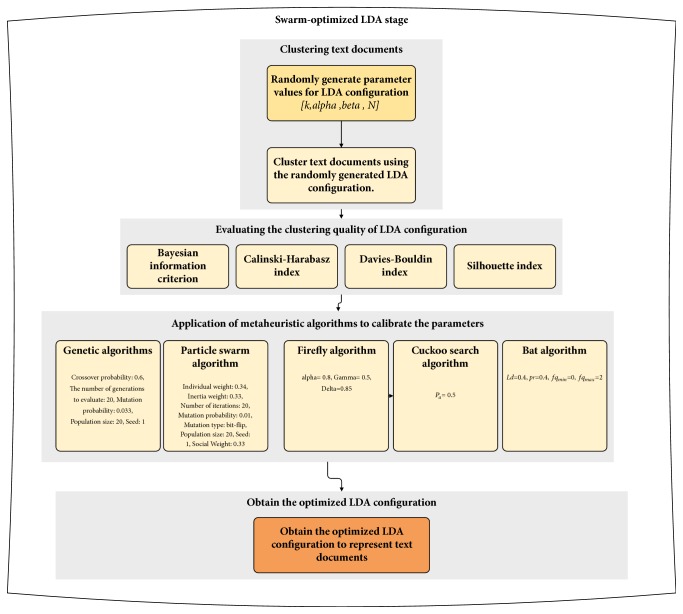
Swarm-optimized latent Dirichlet allocation.

Since exhaustively enumerating possible configurations for LDA can be computationally infeasible task, the identification of a parameter set which maximizes the overall clustering quality can be modelled as an optimization problem. In the presented scheme, five swarm-based optimization algorithms (namely, genetic algorithms, particle swarm optimization, firefly algorithm, cuckoo search algorithm, and bat algorithm) have been considered. The presented approach seeks to find an LDA configuration [*k, α*, *β*,* N*] which maximizes the clustering quality in terms of internal cluster validity indices (Bayesian information criterion, Calinski-Harabasz index, Davies-Bouldin index, and Silhouette index). The presented scheme starts with a randomly generated population of initial configuration. Then, randomly generated LDA configurations are utilized to cluster text documents. The merit of clusters is evaluated using four internal clustering validity indices and the swarm-based optimization algorithms have been utilized to optimize the parameter values. In Figure 2, the general structure of swarm-optimized LDA is summarized.

### 4.2. Diversity-Based Ensemble Pruning

Diversity-based ensemble pruning approach is a hybrid ensemble pruning scheme, which integrates combined pairwise diversity measures and swarm-based clustering algorithms. The presented ensemble pruning method consists of two main stages, namely, computation of pairwise diversity matrices among the base learning algorithms of the ensemble and swarm-based clustering on combined pairwise diversity matrix to obtain final base learning algorithms of the pruned ensemble.

**Figure 3:**
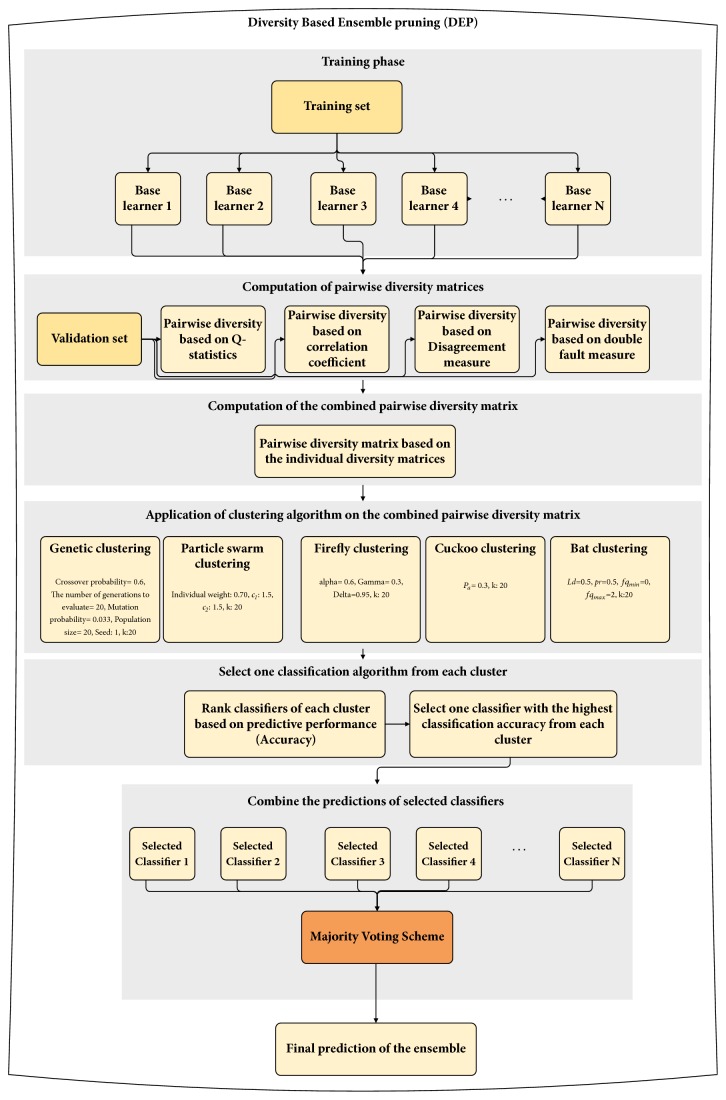
Diversity-based ensemble pruning approach.

The general structure of diversity-based ensemble pruning algorithm is presented in Figure 3. Initially, many different base learning algorithms (classification algorithms) from the model library with varying parameter values have been taken as the initial set of classifiers. The model library contains classification algorithms from five groups, namely, five Bayesian classifiers, fourteen function based classifiers, ten instance based classifiers, three rule based classifiers, and eight decision tree classifier which have been considered. The detailed description regarding the classification algorithms of the model library is presented in Table 2. Classification algorithms of the model library have been trained on the training set. In this way, the predictive characteristics of different learning algorithms have been obtained.

After training classification algorithms, pairwise diversity matrices are computed. The diversity and accuracy are two essential factors to build multiple classifier systems with high predictive performance. There are many pairwise and nonpairwise diversity measures presented in the literature. Different diversity measures concentrate on different aspects of the diversity and there is not a widely accepted definition for the term. Motivated by the success of the combined diversity measures in the ensemble pruning [21], we seek to find an appropriate subset of diversity measures. In this regard, we have conducted an experimental analysis with five widely utilized diversity measures (namely, Q-statistics, correlation coefficient, disagreement measure, double fault measure, and kappa statistics). Since there are five diversity measures, we have evaluated 2^*5*^-1=31 different subset cases. The values obtained for each measure are normalized. Since the highest predictive performance is obtained by averaging the four diversity measures (*Q*-statistics, correlation coefficient, disagreement measure, and double fault measure), this configuration is utilized in the proposed ensemble pruning. For four pairwise diversity measures mentioned above, the diversity values of each pair of classifiers are computed using the validation set. Then, the combined pairwise diversity matrix is obtained from the four pairwise diversity matrices by averaging the diversity values of the individual diversity matrices.

After computation of the combined pairwise diversity matrix, clustering has been employed on the combined diversity matrix. Clustering has been widely employed technique for ensemble pruning, which aims to group classification algorithms into clusters such that the classifiers with the similar characteristics are assigned into the same cluster. By obtaining classifiers from the different clusters, a multiple classifier system with high diversity can be achieved. In this study, five metaheuristic clustering algorithms (namely, genetic algorithm based clustering, particle swarm clustering, firefly clustering, cuckoo clustering, and bat clustering) have been employed on the combined diversity matrix. Based on the clustering results, the classification algorithms have been assigned into a number of clusters.

On the empirical analysis with five metaheuristic clustering algorithms, the highest predictive performance is achieved by firefly clustering algorithm. Hence, we utilized firefly clustering scheme to cluster classification algorithms on the combined diversity matrix based on their predictive characteristics. Let* A *denote an agent that consists of* m n-*dimensional points, *a*_i_ denote* n-*dimensional points in* A*,* P *denote a set containing of* l n*-dimensional points,* p*_*i*_ denote* n*-dimensional point contained in* P,* and* Dist*(*A,P*) denote the distance between* A *and* p*; the general structure of firefly clustering algorithm utilized in the proposed scheme is outlined in Box 2.

After applying clustering algorithm on the combined pairwise diversity matrix, clustering results are utilized to select the classifiers of the pruned ensemble. In order to do so, classifiers of each cluster are ranked based on their predictive performance (in terms of classification accuracy). Then, one classifier with the highest predictive performance is selected from each cluster. Let* N *denote the number of clusters obtained at the end of firefly clustering algorithms, and one classifier has been selected from each classifier. In this way,* N *classifiers constitute the pruned ensemble. In order to combine the predictions of the selected classifiers, majority voting scheme is employed.

## 5. Experimental Analysis

In order to examine the predictive performance of the proposed biomedical text categorization scheme, an extensive empirical analysis has been performed. This section presents the datasets utilized in the analysis, the experimental procedure, and the experimental results.

### 5.1. Dataset

The experimental analysis has been conducted on five public biomedical text categorization datasets. These datasets are Oh5 collection, Oh10 collection, Oh15 collection, Ohscal collection, and Ohsumed-400 collection [77]. Oh5, Oh10, Oh15, Ohscal, and Ohsumed-400 collections are part of OHSUMED collection. Each collection contains biomedical text collections. The basic descriptive information about biomedical text collections utilized in the empirical analysis has been summarized in Table 1, and the number of terms extracted after preprocessing is given.

### 5.2. Evaluation Metrics

In order to evaluate the predictive performance of the presented biomedical text categorization scheme, classification accuracy (ACC) and F-measure have been employed as the evaluation measure.

Classification accuracy is one of the most widely utilized measures in performance evaluation of classification algorithms. It is the proportion of the number of true positives and true negatives obtained by the classifiers in the total number of instances as given by the following equation:

(16) $$\begin{array}{c} ACC=\frac{TN+TP}{TP+FP+FN+TN} \end{array}$$

where* TN, TP, FP, *and* FN *represent the number of true negatives, true positives, false positives, and false negatives, respectively.

F-measure is another common measure in performance evaluation of classification algorithms. F-measure is the harmonic mean of the precision and recall of a classification algorithm. It can take values between 0 and 1 and the higher values of F-measure indicate a better predictive performance. Based on the characteristics of datasets utilized in the empirical analysis, there are two variants of F-measure, namely, micro-averaged F-measure and macro-averaged F-measure. The micro-averaged F-measure extends F-measure to multiclass problems by averaging precision and recall values across all classes. However, F-measure and micro-averaged F-measure cannot focus entirely on rare classes [78]. Since some of the datasets utilized in the empirical analysis are imbalanced dataset, the macro-averaged F-measure is also utilized as another evaluation measure. The macro-averaged F-measure, which determines the average F-measure across all one-versus-all classes, is computed as given by (17):

(17) Macro−averaged  F−measure=1n∑i=1n2×Precisioni×RecalliPrecisioni+Recalli

(18) Precision=TPTP+FP

(19) Recall=TPTP+FN

where* TP, FP, *and* FN *represent the number of true positives, false positives, and false negatives, respectively.

### 5.3. Experimental Procedure

In the experimental analysis, dataset is divided into tenfold (parts). In this scheme, sixfold is utilized for training, twofold is utilized for validation, and twofold is utilized for test. The experimental analysis is performed with the machine learning toolkit WEKA (Waikato Environment for Knowledge Analysis) version 3.9, which is an open-source platform with many machine learning algorithms implemented in Java [79]. The presented classification scheme is also implemented in Java. In the empirical analysis on swarm-based latent Dirichlet allocation, Naïve Bayes algorithm and support vector machines are utilized as the base learning algorithms. In order to compare the presented multiple classifier system, four well-known ensemble methods (namely, AdaBoost, Bagging, Random Subspace, and Stacking) have been considered. For AdaBoost, Bagging, and Random Subspace algorithms, Naïve Bayes and support vector machines are utilized as the base learners. In the Stacking (stacked generalization), the classifier ensemble consisted of five base learners (namely, Naïve Bayes, support vector machines, logistic regression, Bayesian logistic regression, and linear discriminant analysis). For ensemble selection from libraries of models (ESM) and Bagging ensemble selection (BES), the same model library presented in Table 2 has been utilized [19, 20].

For evaluating ensemble pruning schemes, we have adopted the scheme outlined in [19, 20]. In the experimental analysis, ESM, BES, and LibD3C algorithms are considered with different parameter values. For ESM algorithm, four different schemes (namely, forward selection, backward elimination, forward-backward selection, and the best model scheme) have been considered. In ESM algorithm, root mean squared error (RMSE), classification accuracy (ACC), ROC area, precision, recall, and F-measure are considered as the evaluation measures. For BES algorithm, different bag sizes ranging from 10 to 100 are considered. In this algorithm, root mean squared error (RMSE), accuracy (ACC), ROC area, precision, recall, F-measure, and the combination of all metrics are employed as the evaluation measures. For LibD3C algorithm, five different ensemble combination rules (namely, average of probabilities, product of probabilities, majority voting, minimum probability, and maximum probability) are considered. In the experimental analysis, the highest predictive performances obtained from these algorithms are reported. In Table 3, the parameter values of metaheuristic algorithms utilized in swarm-based LDA are presented. In Table 4, parameters of metaheuristic clustering algorithms utilized in the ensemble pruning stage are given. The parameters of the metaheuristic algorithms utilized in the swarm-based LDA stage and the parameters of the metaheuristic algorithms utilized in the ensemble pruning stage are determined based on the benchmark empirical results for the algorithms [80, 81].

### 5.4. Experimental Results and Discussion

The presented biomedical text categorization framework consists of two main stages, namely, swarm-optimized latent Dirichlet allocation stage and diversity-based ensemble pruning stage.

Swarm-optimized latent Dirichlet allocation stage aims to estimate the parameters of LDA. In the empirical analysis on LDA, five different metaheuristic algorithms (namely, genetic algorithms, particle swarm optimization, firefly algorithm, cuckoo search algorithm, and bat algorithm) are considered. To evaluate the clustering quality of different configurations of LDA, four internal cluster validity indices (namely, the Bayesian information criterion, Calinski-Harabasz index, Davies-Bouldin index, and Silhouette index) are considered. In addition, the proposed scheme presents an ensemble pruning based on combined diversity measures and metaheuristic clustering. In the tables, the highest (the best) results achieved by a particular configuration are indicated as both boldface and underline and the second best results are indicated as both boldface and italics.

In order to evaluate the merit of swarm-optimized topic modelling in LDA, Table 5 presents the classification accuracies obtained by different LDA-based configurations with Naïve Bayes and support vector machine classifiers. To verify the impact of ensemble pruning method in the presented scheme, Table 6 presents the classification results obtained by conventional algorithms, ensemble learning methods, conventional ensemble pruning methods, and the proposed diversity-based ensemble pruning method. For the results reported in Table 6, the biomedical text categorization datasets are represented with LDA (*k=*50); i.e., swarm-optimized latent Dirichlet allocation stage has not been applied for the results presented in Table 6 to examine the predictive performance of the proposed ensemble pruning scheme. Finally, Table 7 compares the predictive performance of conventional algorithms, ensemble learning methods, conventional ensemble pruning methods, and the proposed diversity-based ensemble pruning method when swarm-optimized latent Dirichlet allocation stage has been applied to represent the dataset.

As can be observed from the classification accuracies presented in Table 5, the performance of LDA-based representation schemes generally enhances with the use of metaheuristic algorithms in conjunction with LDA to estimate the parameters of it. Among the different metaheuristic algorithms, the highest predictive performance is obtained by bat algorithm based LDA with Davies-Bouldin index based evaluation. The second highest predictive performance is obtained by cuckoo search algorithm based LDA with Davies-Bouldin index based evaluation. Regarding the performance of different evaluation measures, the highest performance is achieved by Davies-Bouldin index based configurations. The second predictive performance is achieved by Silhouette index based configurations, which is followed by Calinski-Harabasz index based configurations. Regarding the performance of conventional LDA-based representation schemes, the highest predictive performance is generally achieved when* k*=50. The predictive performance patterns obtained by different LDA-based configurations with Naïve Bayes algorithm are valid for LDA-based configurations with support vector machines algorithm.

In the empirical analysis on the ensemble pruning, five swarm-based clustering algorithms (namely, genetic clustering, particle swarm-based clustering, firefly clustering, cuckoo clustering, and bat clustering) have been considered. Regarding the predictive performance obtained by conventional classification algorithms, support vector machines algorithm outperforms Naïve Bayes algorithm for the compared datasets. In addition, Bagging ensemble of Naïve Bayes algorithm yields better predictive performance compared to Naïve Bayes algorithm. In general, the predictive performance is enhanced with the use of conventional ensemble learning methods (namely, Bagging, AdaBoost, and Random Subspace algorithm). As can be seen from the results reported in Table 6, conventional ensemble pruning methods outperform the conventional classification algorithms and ensemble learning schemes. In addition, hybrid ensemble pruning schemes (the proposed diversity-based ensemble pruning method, LibD3C algorithm, and ensemble pruning based on combined diversity measures) outperform the other ensemble pruning schemes (ensemble selection from libraries of models and Bagging ensemble selection). The highest predictive performance is obtained by the proposed diversity-based ensemble pruning scheme with firefly clustering. The second highest predictive performance is generally obtained by the proposed diversity-based ensemble pruning scheme with cuckoo clustering.

Based on the extensive empirical analysis with different metaheuristic algorithms in swarm-based LDA and with different clustering algorithms in diversity-based ensemble pruning algorithm, the highest predictive performance is obtained by bat algorithm based LDA with Davies-Bouldin index and diversity-based ensemble pruning with firefly clustering. In Table 7, the predictive performance of the proposed biomedical text categorization scheme is compared with two classification algorithms (namely, Naïve Bayes algorithm and support vector machines), four ensemble methods (namely, Bagging, AdaBoost, Random Subspace, and Stacking), and four ensemble pruning methods (namely, ensemble selection from libraries of models, Bagging ensemble selection, LibD3C algorithm, and ensemble pruning based on combined diversity measures). For the results reported in Table 7, the biomedical text categorization datasets are represented with bat algorithm based LDA with Davies-Bouldin index (BA-LDA (DB)). As can be observed from the results outlined in Table 7, the proposed scheme outperforms the conventional classifiers, ensemble learning methods, and ensemble pruning methods.

In addition to classification accuracy, the predictive performances of classification algorithms, ensemble learning methods, and ensemble pruning methods have been also examined in terms of the macro-averaged F-measure. In Table 8, the macro-averaged F-measure results obtained by different LDA-based configurations with Naïve Bayes and support vector machine classifiers are presented. Regarding the macro-averaged F-measure results presented in Table 8, the highest predictive performance is obtained by bat algorithm based LDA with Davies-Bouldin index based representation. The same patterns obtained in terms of classification accuracies presented in Table 5 are also valid for F-measure based results. Hence, the utilization of metaheuristic optimization algorithms in conjunction with LDA to calibrate its hyper-parameters enhances the predictive model.

To examine the performance improvement achieved by the proposed ensemble pruning scheme, Table 9 presents the macro-averaged F-measure values obtained by conventional algorithms, ensemble learning methods, conventional ensemble pruning methods, and the proposed diversity-based ensemble pruning method. For the results reported in Table 9, the biomedical text categorization datasets are represented with LDA (*k=*50); i.e., swarm-optimized latent Dirichlet allocation stage has not been applied for the results presented in Table 9. Regarding the macro-averaged F-measure results presented in Table 9, the highest predictive performance is obtained by the proposed diversity-based ensemble pruning scheme with firefly clustering. The second highest predictive performance is generally obtained by the proposed diversity-based ensemble pruning scheme with cuckoo clustering and ensemble pruning based on combined diversity.

In Table 10, the macro-averaged F-measure results obtained by classification algorithms, ensemble learning methods, and ensemble pruning methods are presented. For the results reported in Table 10, the biomedical text categorization datasets are represented with bat algorithm based LDA with Davies-Bouldin index (BA-LDA (DB)). Regarding the macro-averaged F-measure results, the proposed scheme outperforms the conventional classifiers, ensemble learning methods, and ensemble pruning methods.

To statistically validate the results obtained in the empirical analysis, we have performed the two-way ANOVA (analysis of variance) test in the Minitab statistical program. The two-way ANOVA test is an extension of the one-way ANOVA test, which aims to evaluate the effect of two different categorical independent variables on one dependent variable. In two-way ANOVA test, both the main effect of each independent variable and their interactions are taken into assessment. The results for the two-way ANOVA test of overall results (in terms of classification accuracy) are presented in Table 11, where DF, SS, MS, F, and P denote degrees of freedom, adjusted sum of squares, adjusted mean square, F-Value, and probability value, respectively. Degrees of freedom are the amount of information in the data. The adjusted sum of squares term (SS) denotes the amount of variation in the response data that is explained by each term of the model. F-statistics (F) is the test statistic to identify whether a term is associated with the response and the probability value (P) is used to determine the statistical significance of the terms and model. The results presented in Table 11 are divided into three parts. The upper part of the table denotes the statistical analysis of results on the different LDA-based configurations, the middle part of the table denotes the statistical analysis of results on ensemble pruning, and the lower part of the table denotes the statistical analysis of results on conventional classifiers, ensemble learning methods, and ensemble pruning methods. For two-way ANOVA test, two different factors (different datasets and different algorithmic configurations) are taken as categorical independent variables. In addition, the interaction among these factors is also taken into consideration. According to the results presented in Table 11, probability value is P<0.001 for different factors and their interactions. Hence, there are statistically meaningful differences between the predictive performances of compared methods. The performance gain obtained by swarm-optimized LDA is statistically meaningful. Similarly, the performance gain obtained by the proposed ensemble pruning method is also statistically meaningful (P<0.001).

The results for the two-way ANOVA test of overall results (in terms of the macro-averaged F-measure values) are presented in Table 12. According to the results presented in Table 12, there are statistically meaningful differences between the predictive performances of compared methods (P<0.001).

**Figure 4:**
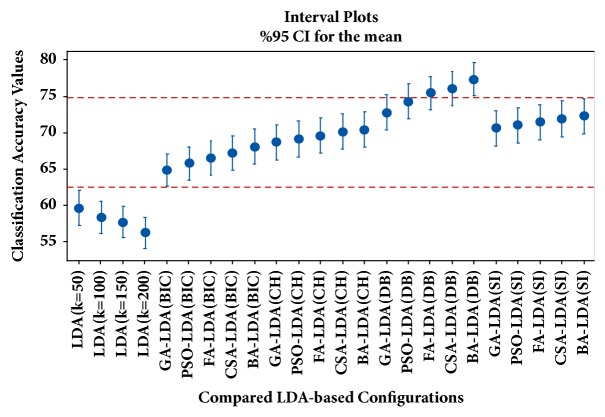
Interval plots for compared LDA-based configurations.

**Figure 5:**
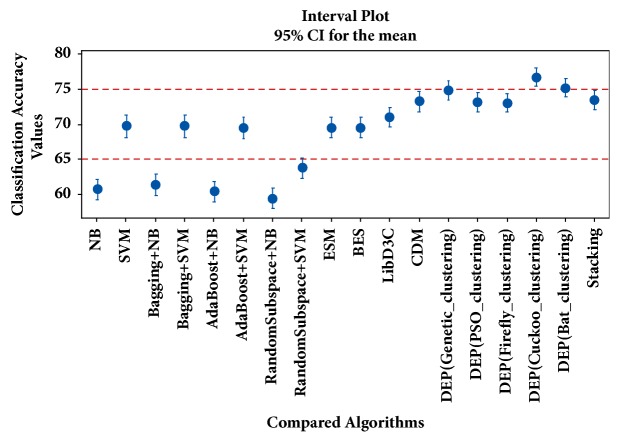
Interval plots for classifiers and ensemble pruning methods.

**Figure 6:**
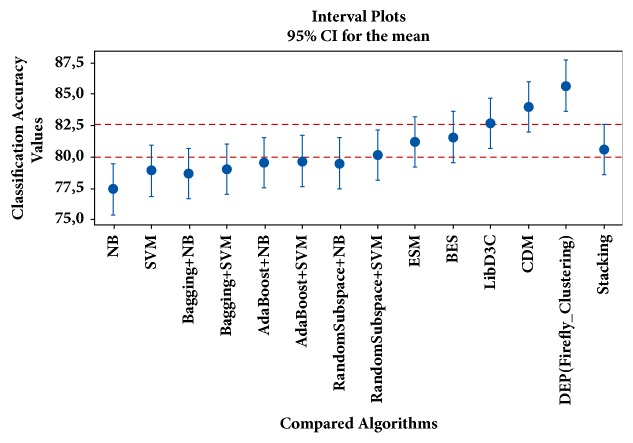
Interval plots for compared algorithms.

In Figure 4, the confidence intervals for the mean values of classification accuracies obtained by the different LDA-based configuration schemes are presented. Similarly, in Figure 5, the confidence intervals for the mean values of classification accuracies obtained by the conventional classifiers, ensemble learners, and ensemble pruning methods are presented. For results depicted in Figure 5, the biomedical text categorization datasets are represented with LDA (*k=*50); i.e., swarm-optimized latent Dirichlet allocation stage has not been applied. In contrast, in Figure 6, the confidence intervals for the mean values of classification accuracies obtained by the conventional classifiers, ensemble learners, and ensemble pruning methods are given. In Figure 6, swarm-optimized latent Dirichlet allocation stage has been applied to represent the dataset. For the statistical significance of results, confidence intervals are divided into regions denoted by red dashed lines. As the interval plots indicate, the predictive performances obtained by the swarm-optimized LDA (BA-LDA (DB)) and DEP (firefly clustering) are statistically significant.

**Figure 7:**
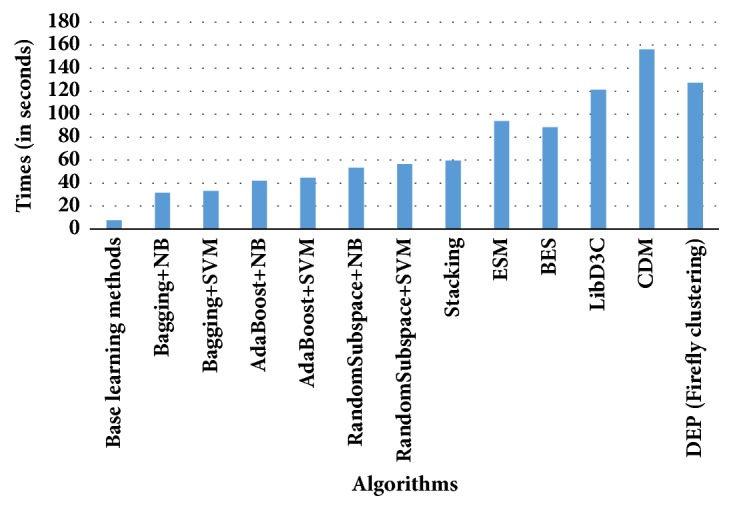
Average execution times (in seconds) for compared algorithms.

In Figure 7, average execution times of compared algorithms have been presented in seconds. As can be observed from Figure 7, average execution times on base learning algorithms (Naïve Bayes and support vector machines) are the lowest. Conventional ensemble learning methods generally enhance the predictive performance of the conventional base learning algorithms. However, ensemble learning methods involve more execution times. Compared to the ensemble learning methods, ensemble pruning schemes have more execution time. The highest execution time is involved in ensemble pruning based on combined diversity measures (CDM) and the second highest execution time is required in the proposed classification scheme (DEP-firefly clustering). Metaheuristic optimization methods are well-established techniques on tuning the parameters. Hence, there is a trade-off between predictive performance and execution times.

## 6. Conclusion

In this work, we propose a novel biomedical text classification scheme based on swarm-optimized latent Dirichlet allocation and diversity-based ensemble pruning. Biomedical text categorization is an important research direction due to the immense quantity of unstructured information available. The latent Dirichlet allocation (LDA) is a popular representation scheme for text documents, which can yield better performance than other linguistic representation schemes, such as latent semantic analysis and probabilistic latent semantic analysis. We found out that the identification of appropriate parameter values is very important to the performance of LDA. In addition, it has been experimentally validated that the use of metaheuristic optimization algorithms to calibrate the parameters of LDA yields promising results on biomedical text categorization. The presented text classification scheme also employs an ensemble pruning approach based on combined diversity measures to identify a robust multiple classifier system with high predictive performance. The presented ensemble pruning approach combines four different diversity measures (namely, disagreement measure,* Q*-statistics, the correlation coefficient, and the double fault measure). In addition, the scheme employs the swarm-based clustering algorithm. The experimental results indicate that the proposed multiple classifier system outperforms the conventional classification algorithms, ensemble learning, and ensemble pruning methods.

## Figures and Tables

**Box 1:**
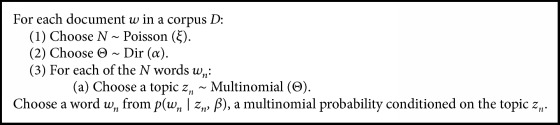
The generative process of LDA (Blei et al., 2013; [19, 20]).

**Box 2:**
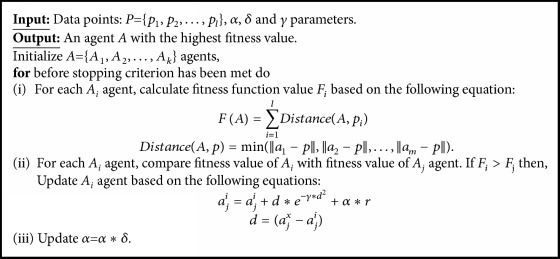
The general structure of firefly clustering algorithm.

**Table 1:** Descriptive information for the datasets.

**Dataset**	**Number of ** **documents**	**Number of ** **terms**	**Average occurrence ** **of terms**	**Number of ** **classes**
Oh5	918	3013	54.43	10
Oh10	1050	3239	55.63	10
Oh15	3101	54142	17.46	10
Ohscal	11162	11466	60.38	10
Ohsumed-400	9200	13512	55.14	12

**Table 2:** Classification algorithms used to build the model library.

**Classifier Group**	**Classification Algorithms**
Bayesian Classifiers (5)	Bayesian logistic regression (with Norm-based hyper-parameter selection), Bayesian logistic regression (with Cross-validated hyper-parameter selection), Bayesian logistic regression (with Specific value based hyper-parameter selection), Naive Bayes, Naive Bayes Multinomial

Function based classifiers (14)	FLDA, Kernel Logistic Regression (with Poly Kernel), Kernel Logistic Regression (with Normalized Poly Kernel), LibLINEAR (with L2-regularized logistic regression), LibLINEAR (with L2-regularized L2-loss support vector classification), LibLINEAR (with L1-regularized logistic regression), LibSVM (with radial basis function), LibSVM (with linear kernel), LibSVM (with polynomial kernel), LibSVM (with sigmoid kernel), Multi-layer perceptron, radial basis function networks, Logistic regression, Gaussian radial basis function networks

Instance based classifiers (10)	KNN (with K: 1), KNN (with K:2), KNN (with K:3), KNN (with K: 4), KNN (with K:5), KNN (with K:6), KNN (with K:7), KNN (with K:8), KNN (with K:9), KNN (with K:10)

Rule based classifiers (3)	FURIA (with Product T-norm), FURIA (with Minimum T-norm), RIPPER

Decision tree classifiers (8)	BFTree (Unpruned), BFTree (Post-pruning), BFTree (Pre-pruning), Functional Tree, C4.5 (J48), NBTree, Random Forest, Random Tree

Table 2 is reproduced from ONAN et al. [19, 20] (under the Creative Commons Attribution License/public domain).

**Table 3:** Parameters of the metaheuristics algorithms utilized in swarm-based LDA.

**Metaheuristic algorithm**	**Parameter Values**
Genetic algorithms	Crossover probability: 0.6, The number of generations to evaluate: 20, Mutation probability: 0.033, Population size: 20, Seed: 1

Particle swarm optimization	Individual weight: 0.34, Inertia weight: 0.33, Number of iterations: 20, Mutation probability: 0.01, Mutation type: bit-flip, Population size: 20, Seed: 1, Social Weight: 0.33

Firefly algorithm	*α*= 0.8, *γ*= 0.5, *δ*=0.85

Cuckoo search algorithm	*P*_*a*_= 0.5

Bat algorithm	*Ld=*0.4, *pr*=0.4, *fq*_*min*_=0, *fq*_*max*_*=*2

**Table 4:** Parameters of the metaheuristics algorithms utilized in ensemble pruning.

**Metaheuristic algorithm**	**Parameter Values**
Genetic clustering	Crossover probability= 0.6, The number of generations to evaluate= 20, Mutation probability= 0.033, Population size= 20, Seed: 1, k:20

Particle swarm clustering	Individual weight: 0.70, *c*_*1*_: 1.5, *c*_*2*_: 1.5, k: 20

Firefly clustering	*α*= 0.6, *γ*= 0.3, *δ*=0.95, k: 20

Cuckoo clustering	*P*_*a*_= 0.3, k: 20

Bat clustering	*Ld=*0.5, *pr*=0.5, *fq*_*min*_=0, *fq*_*max*_*=*2, k:20

**Table 5:** Classification accuracies obtained with different LDA-based configurations.

	**Naive Bayes (NB)**					**Support Vector Machines (SVM)**				
**Configuration**	oh5	oh10	oh15	ohscal	Ohsu-med	oh5	oh10	oh15	ohscal	Ohsu-med

LDA (k=50)	74.38	66.66	69.40	59.27	28.35	76.24	78.73	83.17	70.62	34.64
LDA (k=100)	70.85	63.64	67.44	60.05	29.56	78.28	78.25	83.23	73.23	38.82
LDA (k=150)	69.02	65.24	65.51	59.01	29.43	76.72	79.09	84.74	73.8	41.27
LDA (k=200)	66.17	64.01	63.61	58.93	27.99	77.33	77.93	84	74.19	41.82
GA-LDA (BIC)	75.16	67.24	74.70	71.66	35.45	77.98	69.03	75.12	73.62	35.83
PSO-LDA (BIC)	75.40	68.60	76.90	72.43	35.46	78.22	72.56	75.17	75.89	36.23
FA-LDA (BIC)	75.48	71.26	77.48	72.80	35.60	79.50	74.73	76.63	76.90	37.69
CSA-LDA (BIC)	76.66	71.96	78.77	72.94	35.65	79.56	75.97	77.96	77.02	37.94
BA-LDA (BIC)	78.82	72.21	79.77	73.02	36.58	79.85	76.53	78.89	77.34	38.89
GA-LDA (CH)	79.02	72.88	80.11	74.53	36.85	80.62	77.72	80.31	78.17	38.96
PSO-LDA (CH)	80.20	72.93	80.66	74.76	37.03	81.50	77.91	80.50	78.99	39.03
FA-LDA (CH)	81.20	72.99	80.72	75.13	37.75	81.80	77.99	80.55	79.09	39.03
CSA-LDA (CH)	81.40	73.12	81.71	76.02	38.34	82.61	78.01	80.78	79.82	39.03
BA-LDA (CH)	81.46	73.49	81.82	76.21	39.24	82.87	78.93	81.01	79.89	39.52
GA-LDA (DB)	84.46	76.22	84.13	78.71	40.50	84.73	80.95	85.88	82.46	43.02
PSO-LDA (DB)	84.60	80.07	85.14	79.21	42.57	85.13	81.11	86.17	84.22	43.51
FA-LDA (DB)	85.89	80.82	85.17	80.83	44.60	86.22	81.88	86.73	84.62	44.61
CSA-LDA (DB)	***86.42***	***80.97***	***86.10***	***81.69***	***45.21***	***86.79***	***82.00***	***86.96***	***85.07***	***46.67***
BA-LDA (DB)	**87.60**	**81.36**	**87.32**	**83.56**	**47.00**	**88.86**	**82.09**	**88.05**	**85.24**	**50.08**
GA-LDA (SI)	81.57	73.57	82.03	76.48	39.36	83.21	79.00	82.24	79.93	40.58
PSO-LDA (SI)	82.61	73.76	82.50	76.61	39.66	83.58	79.33	83.03	80.36	40.87
FA-LDA (SI)	83.19	74.18	82.88	77.47	39.68	83.69	79.41	83.11	80.95	40.95
CSA-LDA (SI)	83.78	75.11	83.01	78.06	39.69	83.84	80.83	84.47	81.82	41.12
BA-LDA (SI)	84.11	76.08	83.03	78.13	40.08	84.49	80.90	85.52	81.99	42.65

LDA: latent Dirichlet allocation, GA-LDA: genetic algorithm based LDA, PSO-LDA: particle swarm optimization based LDA, FA-LDA: firefly algorithm based LDA, CSA-LDA: cuckoo search algorithm based LDA, BA-LDA: bat algorithm based LDA, BIC: Bayesian information criterion, CH: Calinski-Harabasz index, DB: Davies-Bouldin index, and SI: Silhouette index.

**Table 6:** Classification results obtained by conventional algorithms and the proposed diversity-based ensemble pruning (with LDA (k=50) based representation).

**Classification algorithm**	**oh5**	**oh10**	**oh15**	**ohscal**	**ohsumed**
NB	75.19	67.43	70.77	60.24	29.41
SVM	77.59	80.29	84.47	71.58	34.72
Bagging+NB	76.08	69.77	70.94	60.21	29.21
Bagging+SVM	84.36	77.20	79.07	71.92	35.98
AdaBoost+NB	73.53	68.07	70.26	60.09	29.60
AdaBoost+SVM	84.06	77.19	78.88	72.08	35.03
RandomSubspace+NB	74.75	67.29	68.51	57.58	28.60
RandomSubspace+SVM	78.02	69.89	71.22	67.65	31.80
Stacking	83.78	81.32	81.69	60.02	40.76
ESM	79.25	79.07	78.91	72.52	37.84
BES	80.11	80.61	81.08	73.02	40.04
LibD3C	82.86	82.93	84.51	74.86	41.17
CDM	84.77	84.13	85.32	76.45	43.55
DEP (Genetic clustering)	81.61	81.96	84.64	74.21	43.27
DEP (PSO clustering)	80.91	81.41	83.31	73.98	***45.73***
DEP (Firefly clustering)	**86.52**	**86.08**	**86.29**	**77.47**	**47.48**
DEP (Cuckoo clustering)	***85.06***	83.00	***85.84***	***76.81***	45.43
DEP (Bat clustering)	84.47	***84.18***	82.11	72.70	44.13

NB: Naïve Bayes algorithm, SVM: support vector machines, ESM: ensemble selection from libraries of models, BES: Bagging ensemble selection, LibD3C: hybrid ensemble pruning based on k-means and dynamic selection, CDM: ensemble pruning based on combined diversity measures, and DEP: the proposed diversity-based ensemble pruning.

**Table 7:** Comparison of the proposed text categorization scheme with conventional classifiers, ensemble learners, and ensemble pruning method (with BA-LDA (DB) based representation).

**Classification algorithm**	**oh5**	**oh10**	**oh15**	**ohscal**	**ohsumed**
NB	87.67	81.42	87.44	83.64	47.09
SVM	88.97	82.22	88.16	85.32	50.08
Bagging+NB	89.32	83.35	88.87	83.47	48.52
Bagging+SVM	88.03	84.84	87.86	83.92	50.73
AdaBoost+NB	89.77	83.60	87.48	86.18	51.18
AdaBoost+SVM	88.18	84.95	87.35	86.29	51.85
RandomSubspace+NB	88.32	83.96	86.66	88.09	50.70
RandomSubspace+SVM	88.56	84.11	89.58	88.29	50.29
Stacking	88.28	86.87	88.93	84.90	53.84
ESM	88.58	86.66	90.25	88.48	51.94
BES	89.29	86.00	90.98	89.12	52.47
LibD3C	90.35	87.95	91.27	90.48	53.41
CDM	***91.51***	***89.61***	***93.17***	***91.33***	***54.47***
Proposed scheme	**93.14**	**91.29**	**93.76**	**92.14**	**58.17**

NB: Naïve Bayes algorithm, SVM: support vector machines, ESM: ensemble selection from libraries of models, BES: Bagging ensemble selection, LibD3C: hybrid ensemble pruning based on k-means and dynamic selection, and CDM: ensemble pruning based on combined diversity measures.

**Table 8:** The macro-averaged F-measure results obtained with different LDA-based configurations.

	**Naive Bayes (NB)**					**Support Vector Machines (SVM)**				
**Configuration**	oh5	oh10	oh15	ohscal	Ohsu-med	oh5	oh10	oh15	ohscal	Ohsu-med

LDA (k=50)	0.75	0.68	0.71	0.61	0.30	0.77	0.80	0.85	0.73	0.36
LDA (k=100)	0.72	0.65	0.69	0.62	0.31	0.79	0.80	0.85	0.75	0.40
LDA (k=150)	0.70	0.67	0.67	0.61	0.31	0.77	0.81	0.86	0.76	0.43
LDA (k=200)	0.67	0.65	0.65	0.61	0.29	0.78	0.80	0.86	0.76	0.44
GA-LDA (BIC)	0.76	0.69	0.76	0.74	0.37	0.79	0.70	0.77	0.76	0.37
PSO-LDA (BIC)	0.76	0.70	0.78	0.75	0.37	0.79	0.74	0.77	0.78	0.38
FA-LDA (BIC)	0.76	0.73	0.79	0.75	0.37	0.80	0.76	0.78	0.79	0.39
CSA-LDA (BIC)	0.77	0.73	0.80	0.75	0.37	0.80	0.78	0.80	0.79	0.40
BA-LDA (BIC)	0.80	0.74	0.81	0.75	0.38	0.81	0.78	0.81	0.80	0.41
GA-LDA (CH)	0.80	0.74	0.82	0.77	0.38	0.81	0.79	0.82	0.81	0.41
PSO-LDA (CH)	0.81	0.74	0.82	0.77	0.39	0.82	0.79	0.82	0.81	0.41
FA-LDA (CH)	0.82	0.74	0.82	0.77	0.39	0.83	0.80	0.82	0.82	0.41
CSA-LDA (CH)	0.82	0.75	0.83	0.78	0.40	0.83	0.80	0.82	0.82	0.41
BA-LDA (CH)	0.82	0.75	0.83	0.79	0.41	0.84	0.81	0.83	0.82	0.41
GA-LDA (DB)	0.85	0.78	0.86	0.81	0.42	0.86	0.83	0.88	0.85	0.45
PSO-LDA (DB)	0.85	***0.82***	0.87	0.82	0.44	0.86	0.83	0.88	0.87	0.45
FA-LDA (DB)	***0.87***	***0.82***	0.87	0.83	0.46	0.87	***0.84***	***0.89***	0.87	0.46
CSA-LDA (DB)	***0.87***	**0.83**	***0.88***	***0.84***	***0.47***	***0.88***	***0.84***	***0.89***	***0.88***	***0.49***
BA-LDA (DB)	**0.88**	**0.83**	**0.89**	**0.86**	**0.49**	**0.90**	**0.84**	**0.90**	**0.88**	**0.52**
GA-LDA (SI)	0.82	0.75	0.84	0.79	0.41	0.84	0.81	0.84	0.82	0.42
PSO-LDA (SI)	0.83	0.75	0.84	0.79	0.41	0.84	0.81	0.85	0.83	0.43
FA-LDA (SI)	0.84	0.76	0.85	0.80	0.41	0.85	0.81	0.85	0.83	0.43
CSA-LDA (SI)	0.85	0.77	0.85	0.80	0.41	0.85	0.82	0.86	0.84	0.43
BA-LDA (SI)	0.85	0.78	0.85	0.81	0.42	0.85	0.83	0.87	0.85	0.44

LDA: latent Dirichlet allocation, GA-LDA: genetic algorithm based LDA, PSO-LDA: particle swarm optimization based LDA, FA-LDA: firefly algorithm based LDA, CSA-LDA: cuckoo search algorithm based LDA, BA-LDA: bat algorithm based LDA, BIC: Bayesian information criterion, CH: Calinski-Harabasz index, DB: Davies-Bouldin index, and SI: Silhouette index.

**Table 9:** The macro-averaged F-measure results obtained by conventional algorithms and the proposed diversity-based ensemble pruning (with LDA (k=50) based representation).

**Classification algorithm**	**oh5**	**oh10**	**oh15**	**ohscal**	**ohsumed**
NB	0.76	0.68	0.72	0.61	0.30
SVM	0.78	0.81	0.86	0.73	0.35
Bagging+NB	0.77	0.70	0.72	0.61	0.30
Bagging+SVM	0.85	0.78	0.81	0.73	0.37
AdaBoost+NB	0.74	0.69	0.72	0.61	0.31
AdaBoost+SVM	0.85	0.78	0.80	0.74	0.36
RandomSubspace+NB	0.76	0.68	0.70	0.59	0.29
RandomSubspace+SVM	0.79	0.71	0.73	0.69	0.33
Stacking	0.84	0.80	0.81	0.72	0.38
ESM	0.80	0.81	0.81	0.74	0.39
BES	0.81	0.82	0.83	0.75	0.41
LibD3C	0.84	0.85	0.86	0.76	0.42
CDM	***0.86***	***0.86***	***0.87***	***0.78***	0.45
DEP (Genetic clustering)	0.82	0.84	0.86	0.76	0.45
DEP (PSO clustering)	0.82	0.83	0.85	0.75	***0.47***
DEP (Firefly clustering)	**0.87**	**0.88**	**0.88**	**0.79**	**0.49**
DEP (Cuckoo clustering)	***0.86***	0.85	**0.88**	***0.78***	***0.47***
DEP (Bat clustering)	0.85	***0.86***	0.84	0.74	0.45

NB: Naïve Bayes algorithm, SVM: support vector machines, ESM: ensemble selection from libraries of models, BES: Bagging ensemble selection, LibD3C: hybrid ensemble pruning based on k-means and dynamic selection, CDM: ensemble pruning based on combined diversity measures, and DEP: the proposed diversity-based ensemble pruning.

**Table 10:** The macro-averaged F-measure results of methods (with BA-LDA (DB) based representation).

**Classification algorithm**	**oh5**	**oh10**	**oh15**	**ohscal**	**ohsumed**
NB	0.89	0.82	0.88	0.84	0.48
SVM	0.90	0.83	0.89	0.86	0.51
Bagging+NB	0.90	0.84	0.90	0.84	0.49
Bagging+SVM	0.89	0.86	0.89	0.85	0.51
AdaBoost+NB	0.91	0.84	0.88	0.87	0.52
AdaBoost+SVM	0.89	0.86	0.88	0.87	0.52
RandomSubspace+NB	0.90	0.86	0.88	0.90	0.52
RandomSubspace+SVM	0.90	0.86	0.91	0.90	0.51
Stacking	0.90	0.87	0.91	0.88	0.54
ESM	0.90	0.88	0.92	0.90	0.53
BES	0.93	0.90	0.95	0.93	0.55
LibD3C	0.94	0.92	0.95	0.94	0.56
CDM	***0.95***	***0.93***	***0.97***	***0.95***	***0.57***
Proposed scheme	**0.97**	**0.95**	**0.98**	**0.96**	**0.61**

NB: Naïve Bayes algorithm, SVM: support vector machines, ESM: ensemble selection from libraries of models, BES: Bagging ensemble selection, LibD3C: hybrid ensemble pruning based on k-means and dynamic selection, and CDM: ensemble pruning based on combined diversity measures.

**Table 11:** Two-way ANOVA test results of classification accuracy values.

**Statistical analysis of results on different LDA-based configurations**					
**Source**	**DF**	**SS**	**MS**	**F**	**P**

Configuration	23	4073.9	177.1	90.50	P<0.001
Dataset	4	60336.7	15084.2	7707.50	P<0.001
Classifier	1	881.0	881.0	450.15	P<0.001
Configuration*∗*Dataset	92	334.0	3.6	1.85	P<0.001
Configuration*∗*Classifier	23	932.9	40.6	20.73	P<0.001
Dataset*∗*Classifier	4	106.3	26.6	13.57	P<0.001
Error	92	180.1	2.0		
Total	239	66844.8			

**Statistical analysis of results on classifiers and ensemble pruning methods (with LDA (k=50) based representation).**					

**Source**	**DF**	**SS**	**MS**	**F**	**P**

Configuration	17	2691.7	158.34	25.86	P<0.001
Dataset	4	23128.7	5782.17	944.48	P<0.001
Error	68	416.3	6.12		
Total	89				

**Statistical analysis of results on conventional classifiers, ensemble learners, and ensemble pruning methods (with BA-LDA (DB) based representation). **					

**Source**	**DF**	**SS**	**MS**	**F**	**P**

Configuration	13	324.5	24.96	17.81	P<0.001
Dataset	4	14736.0	3684.00	2628.98	P<0.001
Error	52	72.9	1.40		
Total	69	15133.4			

**Table 12:** Two-way ANOVA test results of the macro-averaged F-measure.

**Statistical analysis of results on different LDA-based configurations**					
**Source**	**DF**	**SS**	**MS**	**F**	**P**

Configuration	23	0.42777	0.01860	91.27	P<0.001
Dataset	4	5.99867	1.49967	7359.42	P<0.001
Classifier	1	0.09263	0.09263	454.58	P<0.001
Configuration*∗*Dataset	92	0.03536	0.00038	1.89	P<0.001
Configuration*∗*Classifier	23	0.09800	0.00426	20.91	P<0.001
Dataset*∗*Classifier	4	0.01123	0.00281	13.78	P<0.001
Error	92	0.01875	0.00020		
Total	239	6.68241			

**Statistical analysis of results on classifiers and ensemble pruning methods (with LDA (k=50) based representation).**					

**Source**	**DF**	**SS**	**MS**	**F**	**P**

Configuration	17	0.27733	0.016314	23.26	P<0.001
Dataset	4	2.41143	0.692858	859.46	P<0.001
Error	68	0.04770	0.000701		
Total	89	2.73646			

**Statistical analysis of results on conventional classifiers, ensemble learners, and ensemble pruning methods (with BA-LDA (DB) based representation). **					

**Source**	**DF**	**SS**	**MS**	**F**	**P**

Configuration	13	0.03613	0.002780	14.68	P<0.001
Dataset	4	1.53718	0.384296	2029.89	P<0.001
Error	52	0.00984	0.000189		
Total	69	1.58316			

## Data Availability

The data used to support the findings of this study are available from the corresponding author upon request.
